# Nutrient synergy in wheat: Impacts of nitrogen and boron on productivity, accumulation, and soil nutrient retention

**DOI:** 10.1371/journal.pone.0334042

**Published:** 2025-10-06

**Authors:** Prabin Ghimire, Janma Jaya Gairhe, Kishor Kafle, Subodh Khanal

**Affiliations:** 1 Institute of Agriculture and Animal Science, Tribhuvan University, Kathmandu, Nepal; 2 Agriculture and Forestry University, Rampur, Chitwan, Nepal; Nepal Agricultural Research Council, NEPAL

## Abstract

Efficient nutrient management remains a critical challenge in wheat production, particularly in optimizing productivity while maintaining soil fertility. Among essential nutrients, nitrogen (N) and boron (B) play vital roles in plant growth and grain development, yet their combined effects are not fully understood. To address this gap, a field experiment was conducted from November 2019 to April 2020 in Khairahani, Chitwan, Nepal, to evaluate the effects of different N and B application levels on wheat performance and nutrient retention. The experiment followed a split-plot design, with four N levels (0, 80, 100, and 120 kg ha ⁻ ¹) as main plots and three B levels (0, 1, and 2 kg ha ⁻ ¹) as subplots, replicated three times. Results showed that 120 kg ha ⁻ ¹ of N significantly increased effective tillers (401 m ⁻ ²), 1000-grain weight (52.7 g), spike length (10.05 cm), and spike weight (2.3 g). B applied at 2 kg ha ⁻ ¹ reduced spikelet sterility (34.7%) and increased kernels spike^-1^ (29.6). The combined application of N and B produced the highest grain yield (6.26 t ha ⁻ ¹) and benefit-cost ratio (2.34). Grain N content (14.48 g kg ⁻ ¹), N uptake (88.5 kg ha ⁻ ¹), and protein content (9.05%) were maximized with 120 kg N ha ⁻ ¹. Likewise, 2 kg B ha ⁻ ¹ significantly improved B content and uptake in both grain and straw. The nutrient treatments had no significant effect on most soil chemical properties, except for residual soil N and B content. These findings indicate that integrating B with N fertilization can substantially enhance wheat productivity and profitability. However, multi-year studies across diverse agro-climatic conditions are necessary to validate these results and develop robust, site-specific nutrient management recommendations.

## Introduction

Wheat (*Triticum aestivum* L.) is a major staple food crop consumed by over one third of the global population [1], providing nearly 20% of the world’s dietary calorie intake. In 2022, it was grown on approximately 221 million hectares, making it the most widely grown crop globally [2]. In 2023, Nepal produced 2.1 million metric tons of wheat on 0.7 million hectares, with an average yield of 3.01 t ha^-1^ [3]. This yield remains lower than that of neighboring countries like China (5.7 t ha^-1^), India (3.6 t ha^-1^) and Bangladesh (3.5 t ha^-1^) [4,5]. This persistent yield gap reflects low productivity and highlights the urgent need to improve wheat yields through efficient, sustainable agronomic practices [6].

Among various production constraints, poor nutrient management remains a key limiting factors in wheat production. While breeding programs have significantly improved wheat yields [7], insufficient nutrient application continues to limit productivity [8]. Effective nutrient management is essential to maximize yield and minimize nutrient loss [9,10]. Because wheat is nutrient-intensive, balanced application of macro and micronutrients is critical for maintaining soil fertility [11]. Nitrogen (N) is the most limiting macronutrient in wheat production. It plays a critical role by promoting canopy growth and supporting photosynthesis, which ultimately drives yield [12]. Studies confirm that optimal N input enhances biomass, grain yield, and grain quality [13,14]. Boron (B), though required in smaller amounts, is a critical micronutrient for wheat reproduction, influencing processes such as pollen grain development, pollen tube elongation, and grain filling [15–17]. B deficiency is directly associated with increased spikelet sterility and poor yield quality [18], and it is increasingly reported in Nepal due to widespread B-deficient soils [19–21].

Despite these well-documented roles of N and B, their interactive effects under field conditions remain poorly studied. Moreover, there is little information on how B availability modulates the efficiency of N use in B deficient soils. Generalized fertilizer recommendations can limit effectiveness resulting in poor efficiency and low yields. Uneven or inadequate application of nutrients can lower crop productivity and reduce farm profitability, while also increasing the risk of long-term soil degradation and nutrient imbalances [22]. However, the interaction between N and B under field conditions remains underexplored, particularly in B-deficient soils like those in Nepal [23], where localized recommendations and field-based evidence are nonexistent. In addition, factors such as weather conditions, soil physicochemical properties, and fertilizer application methods are critical in agronomic studies, as they strongly influence nutrient availability, plant uptake, and crop responses. Considering these variables provides important context for interpreting field-based nutrient management trials.

To address these gaps, this study investigates the combined effects of N and B on wheat grown in B-deficient soils in Nepal. The specific objectives were to assess how varying levels of N and B affect yield-related traits and overall grain yield; to determine their role in reducing spikelet sterility and improving kernel development; and to examine the nutrient retention content after harvest. We hypothesized that combined N and B application would synergistically improve wheat yield, reproductive traits, and residual soil nutrient levels.

## Materials and methods

### Experimental site

The experiment took place at Khairahani, Chitwan, Nepal which is at 27°36’27.5“N, 84°33’56.2”E, and 168 meters above sea level. This area typically has a rice-based farming system with an average annual rainfall of 232.58 cm and an annual temperature range of 30.79–19.79° C. Experiment was from November 2019 to April 2020. Before the experiment, the topsoil (0–20 cm) from the field was sampled and assessed for its physiochemical properties Table 1 at the Soil and Fertilizer Testing Laboratory, Sundarpur, Kanchanpur, Nepal. The soil is categorized Calcaric Fluvisol (Loamic) according to the World Reference Base for Soil Resources [24].

**Table 1:** Soil physicochemical properties at the experimental site in 2019-2020.

S.N.	Properties	Average content	Methods
1.	Parent Soil	Fluvial, calcareous	Soil map [25]
2.	Physical properties		
	Sand (%)	66.0	
	Silt (%)	27.7	
	Clay (%)	14.3	
	Texture/Rating	Sandy loam	Hydrometer [26]
	Bulk density (g cm^-3^)	1.30	Core [27]
3.	Chemical properties		
	Soil pH	6.94	1:2 soil water [28]
	Soil organic matter (%)	2.38	Walkley &Black [29]
	Total N (%)	0.1	Kjeldahl [30]
	Available phosphorus (kg ha^-1^)	74.62	Modified Olsen bicarbonate method [31]
	Available potassium (kg ha^-1^)	332.6	Ammonium acetate [32]
	Available B (ppm)	0.41	Hot water [33]

### Experimental design and treatment details

The experiment was laid out in a split plot design involving 4 main plots and 3 subplots with 3 replications. The size of the plot was 2m × 2 m (4 m^2^). Each replication was separated by a 1m wide bund and the plot was separated by a 50 cm bund. The crop geometry of wheat was maintained at 25 cm (rows) and continuous (plants).Variety ‘*Vijay*’ was collected from the national wheat research program (NWRP), Rupandehi, Nepal, and selected due to its average maturity period (100–120 days), fertilizer responsiveness, good quality, and low pest infestation [34]. The treatment details of the experimentation is provided in Table 2.

**Table 2:** Treatment details used in the experiment in 2019-2020.

Factor A:Main Plot	Treatments ha^-1^
**N0**	0 kg ha^-1^
**N1**	80 kg ha^-1^
**N2**	100 kg ha^-1^
**N3**	120 kg ha^-1^
**Factor B: Subplot**	
**B0**	0 kg ha^-1^
**B1**	1 kg ha^-1^
**B2**	2 kg ha^-1^

### Crop management

The experimental plots were plowed twice: first, 15 days prior to sowing using a chisel plow, and again one fortnight later using a rotavator to achieve fine tilth. N application was done according to the treatment combinations. N (granulated urea, 48% N) was applied in three splits: 50% as a basal dose at field preparation, 25% at 30 days after sowing (tillering stage), and the remaining 25% at 60 days after sowing (booting stage), following standard recommendations for wheat production in Nepal. B was applied according to treatment (borax, 11% B), phosphorus (50 kg ha ⁻ ¹ as single super phosphate, 16% P₂O₅) and potassium (25 kg ha ⁻ ¹ as muriate of potash, 60% K₂O) as a basal dose, broadcast and incorporated into the soil prior to planting. Seeds were sown manually on 15^th^ November at a depth of 2–3 cm using a seed rate of 120 kg ha ⁻ ¹. No pre-emergence herbicide was applied; instead, weed control was managed through two manual weeding first at the tillering stage (30 days after sowing) and the second 30 days thereafter. One irrigation was applied at the crown root initiation (CRI) stage, equivalent to approximately 60 mm of water (sufficient to restore the soil to field capacity). Subsequent crop water requirements were met by seasonal rainfall during the growing season S1 Table. At physiological maturity (120 days after sowing), plants from the net plot area were harvested manually using sickles.

### Data collection

The number of grains per spike and sterility percentage were determined by counting filled and unfilled grains from 10 sampled spikes. The weight of a thousand grains was measured using an electronic balance from the randomly separated grain yield of the net plot. The sterility percentage was calculated from the ratio of unfilled grains to total grains per spike, which were counted from the 10 spikes sampled [35].


Sterility(%)=(Number of unfilled grains/Total number of grains)X100 (1)

The biomass yield and grain yield were measured from the net plot of 3 rows at harvest. The crop was dried, threshed, and cleaned. The final weight of both straw and grain (before and after drying separately) was recorded. The net plot yield was used to calculate the grain yield per hectare for each treatment.


$$\text{Grain yield}(\text{kg h}{\text{a}}^{-\text{1}})\text{at 14}\%\text{moisture}=((\text{100}-\text{MC})\text{x plot yield}(\text{Kg})\text{x 10000})/((\text{100}-\text{14})\text{x net plot area})$$ (2)

Where MC is the grain moisture percentage. The straw yield from the net plot rows was measured and converted to a hectare.

### Plant sample analysis

Grain samples were oven-dried at 65 °C until they reached a constant weight, typically requiring approximately three days. Fresh weights were recorded immediately after collection to avoid moisture loss. For straw, subsamples (200–300 g) were similarly dried at 65 °C until a stable weight was achieved to ensure accurate estimation of dry matter content. Grinder machines were used to grind both oven-dried grain and straw samples, and the resulting material was stored in a brown paper bag for subsequent nutrient content analysis in a laboratory, following standard procedures. To determine the total N content, the micro Kjeldahl method, involving a mixture of concentrated sulfuric acid and digestion reagents, was employed for the digestion of straw and grain samples [30]. The hot-water extractable method was applied to both straw and grain samples [33].

### Nutrient uptake (NU) and grain protein concentration (GPC)

Nutrient uptake in both grain and straw was calculated based on nutrient concentration and the corresponding oven-dry biomass yield. Nutrient concentration in the samples was determined on a dry-weight basis. The nutrient uptake was then calculated using the following formula:


$$\text{N}{\text{U}}_{\left(\text{grain}\right)}(\text{kg h}{\text{a}}^{-\text{1}})=((\text{Nutrient concentration on grain}\times \text{Oven dry weight of grain})/\text{100})$$ (3)

$$\text{N}{\text{U}}_{\left(\text{Straw}\right)}(\text{kg h}{\text{a}}^{-\text{1}})=((\text{Nutrient concentration on straw}\times \text{Oven dry weight of straw})/\text{100})$$ (4)

GPC(%)=Grain N(%)×6.25 (5)

### Post-harvest soil analysis

Residual soil N and B contents were analyzed from soil samples collected at a depth of 0–20 cm after harvest. From each experimental plot, five subsamples were collected in a zigzag pattern using a soil auger and subsequently mixed thoroughly to form a composite sample. The composite samples were then air-dried, sieved through a 2 mm mesh, and analyzed for nutrient content following the procedures described in Table 1.

### Economic analysis

The cost of cultivation (NRs. ha^-1^) was calculated based on the local charges for different agro-inputs. The economic yield of wheat was converted into gross return (NRs. ha^-1^) based on local market price. The benefit-to-cost (B:C) ratio was calculated. For international reference, the average exchange rate during manuscript preparation (June 2025) was approximately 1 USD = 137.21 NPR.


B:C ratio=Gross return/Cost of cultivation. (6)

### Statistical analysis

All statistical analyses were conducted using R software (version 4.2). A split-plot design was used with N levels as the main plot and B levels as the subplot treatments. A linear mixed-effects model (LMM) was fitted using the lmer() function from the lme4 package, with fixed effects of N, B, and their interaction (N × B), and replication and replication × N interaction as random effects. Type III ANOVA was performed using the car package to determine the significance of treatment effects. Estimated marginal means (EMMs) were calculated using the emmeans package, and Tukey’s honest significant difference (HSD) test was used for multiple comparisons. Significant differences between means were separated using compact letter displays (CLD). All visualizations were created using ggplot2, plots were annotated with statistical groupings. A significance level of *p* < 0.05 was used throughout the analysis.

Model equation:


$${\text{Y}}_{\text{ijk}}=\mu +A_{i}+B_{j}+{\left(AB\right)}_{ij}+\text{Rk}+{\left(AR\right)}_{ik}+\epsilon_{ijk}$$ (7)

Where:

- Y_ijkY_ is the observed response variable
- μ is the overall mean
- A_i_ is the fixed effect of N level
- B_j_ is the fixed effect of B level
- (AB)_ij_ is the interaction effect between N and B
- R_k_ is the random effect of replication
- (AR)_ik_ is the random interaction of replication × N
- ε_ijk_ is the residual error term

## Results

### Yield attributing characteristics of wheat

N application at N3 significantly increased 1000 grain weight by 19.2% (*p* < 0.001), compared to control (N0) Table 3. Spike length and spike weight were also significantly enhanced by 26.7% (**p* *< 0.05) and 17.6% (*p* < 0.001), respectively, under N3 as compared to control Table 3. B application significantly reduced sterility by 37.0% (*p* < 0.001), increased grain spike^-1^ by 31.6% (*p* < 0.001) and spike weight increased by 6.7% with increasing B levels (*p* < 0.05) in 2 kg ha^-1^ as compared to control (B0) Table 3. A significant N × B interaction (*p* < 0.001) was observed only for spike weight, highest value was recorded at N and B application at the rate of N3 and B2 respectively S2 Table.

**Table 3:** Effect of different levels N and B on yield attributing characteristics of wheat at Khairahani, Chitwan, Nepal.

Treatments	Effective tillers m^-2^	Sterility (%)	Grain spike^-1^	1000-grain weight (g)	Spike length (cm)	Spike weight (g)
**Main-plots**						
**N0**	271 c	44	24.8	44.2 b	7.93 c	1.99 d
**N1**	330 b	43.2	28	46.4 b	9.01 b	2.06 c
**N2**	354 b	42.9	26	47 b	9.58 ab	2.22 b
**N3**	401 a	44.8	26	52.7 a	10.05 a	2.34 a
**F test (P > F)**	**	NS	NS	***	*	***
**HSD (0.05)**	44.83	6.942	4.7	3.743	0.739	0.037
**SEM ±**	14.8	2.51	1.71	0.77	1.95	0.013
**Sub-plots**						
**B0**	337	55.1 a	22.5 b	48.4	9.07	2.08 c
**B1**	339	41.4 b	27.1 a	47.4	9.08	2.16 b
**B2**	340	34.7 c	29.6 a	47	9.02	2.22 a
**F test (P > F)**	NS	***	***	NS	NS	*
**HSD (0.05)**	40.54	6.277	4.25	3.38	0.669	0.033
**SEM ±**	14	1.61	1.22	0.67	0.179	0.008
**Interactions**						
**F test (P > F)**	NS	NS	NS	NS	NS	***
**HSD (0.05)**	58.81	9.105	6.17	4.9	0.97	0.048
**SEM ±**	19.8	4.57	2.19	1.34	0.292	0.017

***, **, and * indicate statistical significance at the 0.001,0.01 and 0.05 levels, respectively. Means followed by the same letter(s) within a column are not significantly different according to Tukey’s post hoc test at the 5% level. NS = non-significant; SEM = standard error of the mean; HSD = honest significant difference.

### Grain yield and straw dry matter of wheat

**Fig 1:**
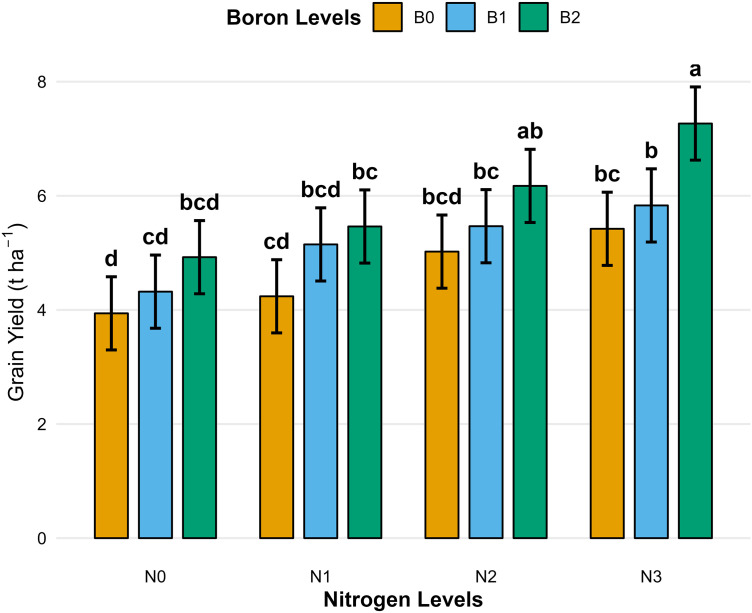
Grain yield (t ha^-1^) of wheat. Different lowercase letter indicate significant difference at *p* < 0.05. Bar indicate a standard error.

N3 significantly enhanced grain yield by 40.6% (*p* < 0.001), straw dry matter accumulation by 32.6% (*p* < 0.01), compared to the control (N0) Table 4. B application also had a significant effect, increasing grain yield by 27.9% (*p* < 0.05) under B2 (2 kg ha ⁻ ¹) compared to B0 Table 4. A significant N × B interaction was observed for grain yield (*p* < 0.01; Fig 1) with the highest values recorded under the combined application of N3 and B2.

**Table 4:** Effect of different levels N and B on yields of wheat at Khairahani, Chitwan, Nepal (2019-2020).

Treatments	Grain yield (t ha^-1^)	Straw dry matter (t ha^-1^)
**Mainplots**		
**N0**	4.39 c	5.49 b
**N1**	4.95 bc	6.19 ab
**N2**	5.55 ab	7.08 a
**N3**	6.17 a	7.28 a
**F test (P > F)**	***	**
**HSD (0.05)**	0.73	1.55
**SEM ±**	0.6	0.81
**Subplots**		
**B0**	4.66 c	6.32
**B1**	5.19 b	6.41
**B2**	5.96 a	6.76
**F test (P > F)**	*	NS
**HSD(0.05)**	0.66	1.4
**SEM ±**	0.59	0.79
**Interaction**		
**F test (P > F)**	*	NS
**HSD(0.05)**	0.96	2.03
**SEM ±**	0.64	0.92

***, **, and * indicate statistical significance at the 0.001, 0.01, and 0.05 levels, respectively. Means followed by the same letter(s) within a column are not significantly different according to Tukey’s post hoc test at the 5% level. NS = non-significant; SEM = standard error of the mean; HSD = honest significant difference.

### N content and uptake at harvest of wheat

N application significantly increased N concentration and uptake in both grain and straw (*p* < 0.001) Table 5. Grain N content increased by 69.7%, grain protein content was increase by 56.5% while grain N uptake increased by 137.2% under N3 as compared to N0. Similarly, straw N content increased by 109.3% and straw N uptake by 181.4% in N3 as compared to N0 Table 5. B application had a significant effect only on grain N uptake (*p* < 0.05), which increased by 26.9% from 56.5 kg ha^-1^ under B0 to 71.7 kg ha^-1^ under B2 Table 5. No significant effects of B were observed on grain or straw N concentration, nor on straw N uptake. No significant interaction effects between N and B treatments were observed for any of the N related parameters measured Table 5.

**Table 5:** Effect of different levels N and B on grain and straw N content and uptake of wheat at Khairahani, Chitwan, Nepal (2019-2020).

Treatments	Grain N content (g kg^-1^)	Grain protein (%)	Grain N uptake (kg ha^-1^)	Straw N content (g kg^-1^)	Straw N uptake (kg ha^-1^)
**Mainplots**					
**N0**	8.53 c	5.33 c	37.3 c	2.36 c	12.9 c
**N1**	11.44 b	7.15 b	57.0 b	2.53 c	15.8 c
**N2**	13.34 a	8.34 a	74.0 a	3.18 b	22.6 b
**N3**	14.48 a	9.05 a	88.5 a	4.94 a	36.3 a
**F test (P > F)**	***	***	***	***	***
**HSD(0.05)**	1.58	1.58	15.08	0.57	6.6
**SEM ±**	0.34	0.21	6.85	0.28	4.04
**Subplots**					
**B0**	12.0	7.47	56.5b	3.12	22.2
**B1**	12.1	7.58	64.5 ab	3.38	22.0
**B2**	11.8	7.34	71.7 a	3.26	21.5
**F test (P > F)**	NS	NS	*	NS	NS
**HSD(0.05)**	1.43	1.43	13.63	0.56	6.44
**SEM ±**	0.29	0.18	6.67	0.19	3.68
**Interactions**					
**F test (P > F)**	NS	NS	NS	NS	NS
**HSD(0.05)**	2.08	2.08	19.78	0.82	9.34
**SEM ±**	0.57	0.36	8.15	0.33	4.54

***, and * indicate statistical significance at the 0.001, and 0.05 levels, respectively. Means followed by the same letter(s) within a column are not significantly different according to Tukey’s post hoc test at the 5% level. NS = non-significant; SEM = standard error of the mean; HSD = honest significant difference.

### B content and uptake at harvest of wheat

B application at B2 significantly enhanced grain B content by 169% (**p* *< 0.01) and straw B content by 9.3% (*p* < 0.001) relative to B0. Grain B uptake increased by 33.7% (*p* < 0.05), and straw B uptake increased by 30.5% (*p* < 0.001) under B2 as compared to B0 Table 6, whereas interaction effects for all other traits were not statistically significant (*p* > 0.05) Table 6.

**Table 6:** Effect of different levels N and B on grain and straw B content and uptake of wheat at Khairahani, Chitwan, Nepal (2019-2020).

Treatments	Grain B (mg kg^-1^)	Straw B (mg kg^-1^)	Grain B uptake (kg ha^-1^) X 10^−5^	Straw B uptake(kg ha^-1^) X 10^−5^
**Mainplots**				
**N0**	3.82	12.3	1.72	6.76
**N1**	3.75	12.4	1.92	7.67
**N2**	3.80	12.7	2.16	8.96
**N3**	3.58	12.2	2.30	8.82
**F test (P > F)**	NS	NS	NS	NS
**HSD(0.05)**	0.56	0.45	0.6	2.21
**SEM ±**	0.163	0.12	0.24	0.11
**Subplots**				
**B0**	1.87 c	11.8 c	0.85 c	6.92 c
**B1**	4.31 b	12.5 b	2.23 b	7.96 b
**B2**	5.03 a	12.9 a	2.99 a	8.29 a
**F test (P > F)**	**	***	*	***
**HSD(0.05)**	0.51	0.41	0.5	0.32
**SEM ±**	0.152	0.09	0.24	0.09
**Interactions**				
**F test (P > F)**	NS	NS	NS	NS
**HSD(0.05)**	0.74	0.59	0.7	1.84
**SEM ±**	0.233	0.18	0.29	1.16

***, **, and * indicate statistical significance at the 0.001, 0.01, and 0.05 levels, respectively. Means followed by the same letter(s) within a column are not significantly different according to Tukey’s post hoc test at the 5% level. NS = non-significant; SEM = standard error of the mean; HSD = honest significant difference.

### Economics of wheat production

**Fig 2:**
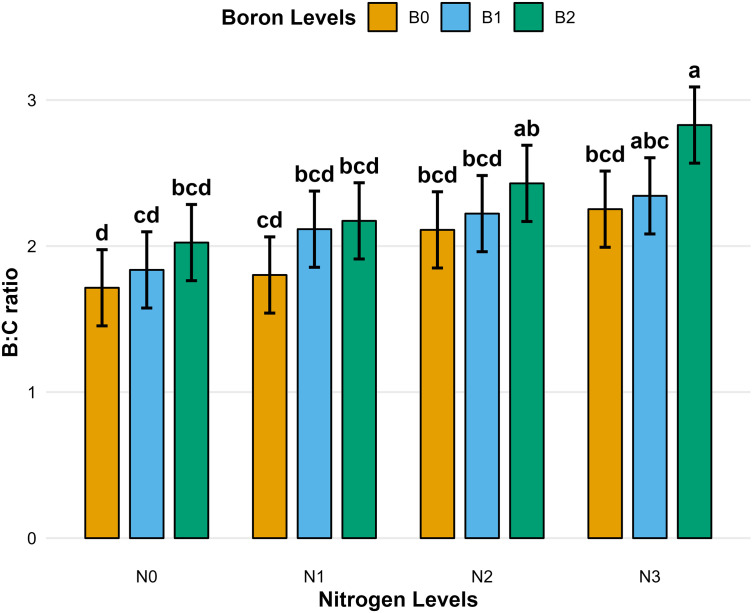
BC ratio of wheat production. Different lowercase letter indicate significant difference at *p* < 0.05. Bar indicate a standard error.

Application of N significantly influenced all economic parameters of wheat production (*p* < 0.05). Gross returns increased by 39.9% under N3 compared to N0, while net returns improved by 81.4%. The benefit-cost (B:C) ratio also increased from 1.86 under N0 to 2.48 under N3, representing a 33.3% improvement (*p* < 0.001). Similarly, B application significantly affected gross returns (*p* < 0.05), with B2 resulting in a 27.9% increase compared to B0 Table 7. Although differences in net returns and B:C ratio across B levels were not statistically significant (*p* > 0.05), a numerical improvement was observed from B0 to B2. A significant N × B interaction (*p* < 0.001) was detected for all economic traits Table 7 (Fig 2).

**Table 7:** Effect of different levels N and B on economics of wheat production at Khairahani, Chitwan, Nepal (2019-2020).

Treatments	Cost of production (000 NPR ha^-1^)	Gross returns (000 NPR ha^-1^)	Net Returns (000 NPR ha^-1^)	B:C ratio
**Mainplots**				
**N0**	87.4	163 c	75.2 c	1.86 c
**N1**	90	183 bc	93.1 bc	2.03 bc
**N2**	91	206 ab	114.5 ab	2.25 ab
**N3**	92	228 a	136.4 a	2.48 a
**F test (P > F)**		*	***	***
**HSD(0.05)**		27.32	27.32	0.28
**SEM ±**		22.3	22.3	0.24
**Subplots**				
**B0**	87.3	172 c	85	1.97
**B1**	90	192 b	102	2.13
**B2**	93.0	220 c	127	2.36
**F test (P > F)**		*	NS	NS
**HSD(0.05)**		24.71	24.71	0.25
**SEM ±**		22.1	22.1	0.24
**Interaction**				
**F test (P > F)**		***	***	***
**HSD(0.05)**		35.84	30.84	0.37
**SEM ±**		23.7	23.7	0.26

***, and * indicate statistical significance at the 0.001, and 0.05 levels, respectively. Means followed by the same letter(s) within a column are not significantly different according to Tukey’s post hoc test at the 5% level. NS = non-significant; SEM = standard error of the mean; HSD = honest significant difference.

### Soil chemical properties after harvest

Post-harvest soil analysis revealed that N application significantly influenced soil total N content (*p* < 0.01), while its effects on pH, organic matter (OM), available phosphorus (P₂O₅), potassium (K₂O), and B were not statistically significant (*p* > 0.05) Table 8. Total N increased progressively with higher N application, reaching a maximum of 0.148% under N3 compared to 0.067% under N0 led to a 120.9% increase. Soil B significantly increased by 92.8% under B1 (0.32 mg kg ⁻ ¹) and by 267.5% under B2 (0.61 mg kg ⁻ ¹) Table 8. However, other parameters such as soil pH, OM, P₂O₅, and K₂O were unaffected by B levels (*p* > 0.05). No significant N × B interaction was detected for any of the measured soil properties (*p* > 0.05), indicating that the combined application of N and B did not produce synergistic or antagonistic effects on soil chemical status.

**Table 8:** Effect of different levels N and B on post harvest soil properties at Khairahani, Chitwan, Nepal (2019-2020).

Treatments	N (%)	pH	OM (%)	P_2_O_5_ (kg ha^-1^)	K_2_O (kg ha^-1^)	B (mg kg^-1^)
**Mainplots**						
**N0**	0.067 c	6.36	2.15	76.66	304.25	0.348
**N1**	0.088 c	6.27	2.19	77.6	293.65	0.363
**N2**	0.115 bc	6.1	2.22	78.04	336.19	0.336
**N3**	0.148 a	6.21	2.17	75.53	333.58	0.41
**F test (P > F)**	**	NS	NS	NS	NS	NS
**HSD(0.05)**	0.022	0.73	1.509	17.108	117.237	0.167
**SEM ±**	0.0136	0.158	0.122	1.76	24.3	0.05
**Subplots**						
**B0**	0.0917	6.13	2.11	71.7	320.45	0.166 c
**B1**	0.11	6.15	2.14	73.7	324.44	0.32 b
**B2**	0.114	6.43	2.18	72.4	305.45	0.61 a
**F test (P > F)**	NS	NS	NS	NS	NS	***
**HSD(0.05)**	0.02	0.66	1.46	16.47	106.107	0.151
**SEM ±**	0.009	0.133	0.105	1.52	21.54	0.04
**Interaction**						
**F test (P > F)**	NS	NS	NS	NS	NS	NS
**HSD(0.05)**	0.029	0.958	1.667	19.322	153.772	0.219
**SEM ±**	0.015	0.266	0.193	2.73	42.1	0.06

***, and ** indicate statistical significance at the 0.001, and 0.01 levels, respectively. Means followed by the same letter(s) within a column are not significantly different according to Tukey’s post hoc test at the 5% level. NS = non-significant; SEM = standard error of the mean; HSD = honest significant difference.

## Discussion

The results demonstrated that integrated application of N and B significantly enhanced grain yield, improved yield-attributing traits, reduced sterility, and increased nutrient uptake, with notable N × B interactions observed for several agronomic and economic parameters. These findings are consistent with our hypothesis that the combined application of N and B would synergistically enhance wheat productivity.

The wheat yield greatly depends on the supply of chemical fertilizer, particularly N [36–40]. In our study, N application treatment significantly increased the grain yield and total dry matter accumulation of wheat as compared to N0. N application enhances chlorophyll content and CO₂ assimilation rate in crops [41], thereby promoting aboveground biomass production and its translocation to grains, critical factors for improving yield [42,43]. This aligns with the physiological role of N in enhancing the source–sink relationship in wheat, thereby increasing biomass and N uptake [44]. Consistent with earlier findings [13,17,37], wheat grain yield and straw dry matter increased significantly with N application, with optimal performance observed around 120–125 kg N ha ⁻ ¹ in a similar Nepalese context.

However, wheat yield is a complex trait determined by spike number m^-2^, kernel spike^-1^, and 1000-grain weight [45,46]. We found that N application significantly increased the spike number m^-2^, 1000- grain weight, while B application reduced sterility % and increase the kernel spike^-1^ resulting in significant differences in yield. Tiller fertility is a key factor in determining grain yield and is highly responsive to N fertilization [47]. Applying optimum N is crucial for balancing tiller production and productivity in wheat. It supports the development of effective tillers while minimizing the formation of non-productive ones, thereby enhancing overall yield without causing excessive competition among plants [48,49]. Previous findings showed that N fertilizer application significantly improved wheat performance compared to the control, with increases of 42.6% in grain weight ear^-1^, 26.8% in grains spike^-1^, 15.4% in 1000-grain weight, and 16.0% in overall yield [50]. Similarly, Liu et al. [51] also found the 1000-grain weight and spike weight increases with increasing N. Additionally application of N significantly increased the spike length and spike weight compared to the control [52]. Furthermore, sterility, filled grain per spike and spike weight were greatly influenced by B application too, particularly filled grain spike^-1^ and spike weight were significantly increased while sterility decreased with B application as compared to control highlighting its essential role in wheat fertility, particularly in pollen viability and fertilization thereby serving as a remarkable indicator of the substantial potential for increased yield in wheat [53]. Availability of B during microsporogenesis is crucial, which impacts anther development, pollen viability and germination, as well as pollen tube growth, ultimately leading to male fertility and increased kernel spike^-1^ [54]. Similarly, several studies have demonstrated that B application can enhance both the yield and quality of crops, Ahmad et al. [55] recommended applying up to 2.5 kg B ha ⁻ ¹ to address B deficiencies in major crops. Moreover, combined application of N and B in winter triticale increased the yield [56]. The weather during the cropping season was generally favorable, with moderate temperatures and timely rainfall between December and March ensuring adequate soil moisture at critical growth stages. Although weather data were not statistically modeled in this study, these conditions likely supported high yield potential and enhanced crop responsiveness to the applied treatments.

The plant N concentration is a key indicator of N limitation in crops and is positively associated with productivity under N-limited conditions [57,58]. Application of N fertilizer enhances soil N availability [58], thereby improving plant N uptake [59]. In this study the concentration and uptake of N is both grain and straw increased significantly with N application, which is consistent with the result of [60]. This could be due to enhanced soil N availability and improved plant uptake efficiency [61]. Greater N supply also supports higher biomass production and better translocation of N to the grain, resulting in increased accumulation in both grain and straw [62].

Based on findings of the study [63], N uptake in wheat increased progressively from tillering to maturity, driven by both greater dry matter production and higher N concentration. Increasing N application from 100 to 150 kg ha ⁻ ¹ significantly enhanced N accumulation in both grain and straw, highlighting the strong relationship between N supply and nutrient accumulation efficiency in wheat. Moreover a study in Nepal has resulted the two year mean accumulation of N in wheat grain was observed to be 86.8 kg ha^-1^ when N is applied at 125 kg ha^-1^ [37].

Similarly, various previous researchers reached a similar conclusion, which emphasized the management of N to improve the concentration and uptake in both grain and straw [60,64,65].

In our study, we found B concentration and uptake is significant to applied B in both grain and straw, similar to the [66] who found that there was an increasing trends for B concentration with increasing B levels in wheat. Furthermore, our results is consistent with [67] who found B concentration and uptake increase with B application. Similar to our result the concentration of B is low in grain as compared to straw [66], because B has limited phloem mobility and grains have low transpiration, making it difficult for B to be transported and accumulated in grains [68,69].

The significant increase in residual soil N and B observed in our study can be attributed to the direct addition of these nutrients through fertilizer application. Numerous studies have shown that long-term fertilizer application enhances soil nutrient reserves, thereby increasing plant-available N in the soil [70,71] while [72] demonstrated that repeated N fertilizer application significantly increased soil NO₃ ⁻ –N concentrations, crop yield, and N accumulation in wheat for up to two years after fertilization had ceased. These findings underscore the persistence of residual N in the soil even after the last fertilizer input, especially when crop uptake is limited or environmental conditions favor N retention. Our study, although conducted over a shorter term, also showed elevated residual N and B in the soil, supporting the idea that nutrient accumulation is not solely a long-term process but can occur relatively quickly under favorable soil and climatic conditions. Consistent with this, [73] observed that low nutrient uptake efficiency often results in higher soil NO₃ ⁻ –N post-harvest, highlighting the importance of aligning fertilizer supply with crop demand to avoid nutrient surplus. A study conducted in Nepal on the residual effect of B in maize reported pronounced benefits on subsequent crops even without additional applications. However, the magnitude of these residual effects was largely influenced by soil texture and type [74].The potential role of soil texture in our case (sandy loam) soils in nutrient leaching was not assessed in this study, although the literature suggests that coarser-textured soils are more prone to such losses. The slightly acidic soil pH likely promoted B availability in the soil solution, thereby facilitating greater uptake by wheat plants [75]. Similarly, a study reported that the pronounced effect of B on yield was observed in lowland areas, mainly due to higher relative humidity, which influences B translocation [76]. These baseline conditions partly explain the crop responses observed to N and B. While these findings are insightful, we are not making any definitive recommendations regarding changes in soil physicochemical properties. Instead, we emphasize the need for long-term trials to better understand the residual effects of continuous nutrient application on soil health and fertility dynamics.

## Conclusions

This study demonstrated that the combined application of N and B significantly enhanced wheat yield, yield-attributing traits, and profitability. Additionally, N and B applications significantly increased their respective residual levels in the soil without adverse effects on other soil properties which suggests annual applications B fertilization appears unnecessary in wheat-based systems of Nepal. By simultaneously examining both yield responses and post-harvest soil nutrient status, this study offers a novel and integrative perspective on the role of B in enhancing wheat performance under field conditions, a combination that has received limited attention in South Asian wheat cropping systems.

However, as this was a single-year field study, the results should be interpreted with caution. Multi-year experiments involving a broader range of N and B application rates, diverse wheat genotypes, and varied agroclimatic conditions are necessary to validate and generalize the findings. Nonetheless, this research provides an important starting point for understanding the role of B in wheat production and offers valuable insights for developing integrated nutrient management strategies. Future research should focus on genotype-specific N × B responses, multi-year experiments across diverse agro-climatic regions of Nepal to validate these findings and assess long-term effects of N and B fertilization on soil fertility.

## Supporting information

S1 Fig Climatic condition during the experimentation period at Khairahani, Chitwan, Nepal. (TIF)

S1 Table Monthly weather data for the study area from November 2019 to December 2020. The table includes average maximum and minimum air temperature (°C), total rainfall (mm), and total evaporation (mm) recorded for each month.
(DOCX)

S2 Table Summary statistics (mean, standard deviation, and standard error) of yields attributing traits under different N and B treatments. (DOCX)

S3 Table Summary statistics (mean, standard deviation, and standard error) of yields under different N and B treatments. (DOCX)

S4 Table Summary statistics (mean, standard deviation, and standard error) of N content and uptake in grain and straw under different N and B treatments. (DOCX)

S5 Table Summary statistics (mean, standard deviation, and standard error) of B content and uptake in grain and straw under different N and B treatments. (DOCX)

S6 Table Summary statistics (mean, standard deviation, and standard error) of post harvest soil properties under different N and B treatments. (DOCX)
