# Supplementary figures and images for: Nutrient synergy in wheat: Impacts of nitrogen and boron on productivity, accumulation, and soil nutrient retention
Source: PLoS One. 2025 Oct 6;20(10):e0334042. doi: 10.1371/journal.pone.0334042 (PMC12500113; doi:10.1371/journal.pone.0334042)

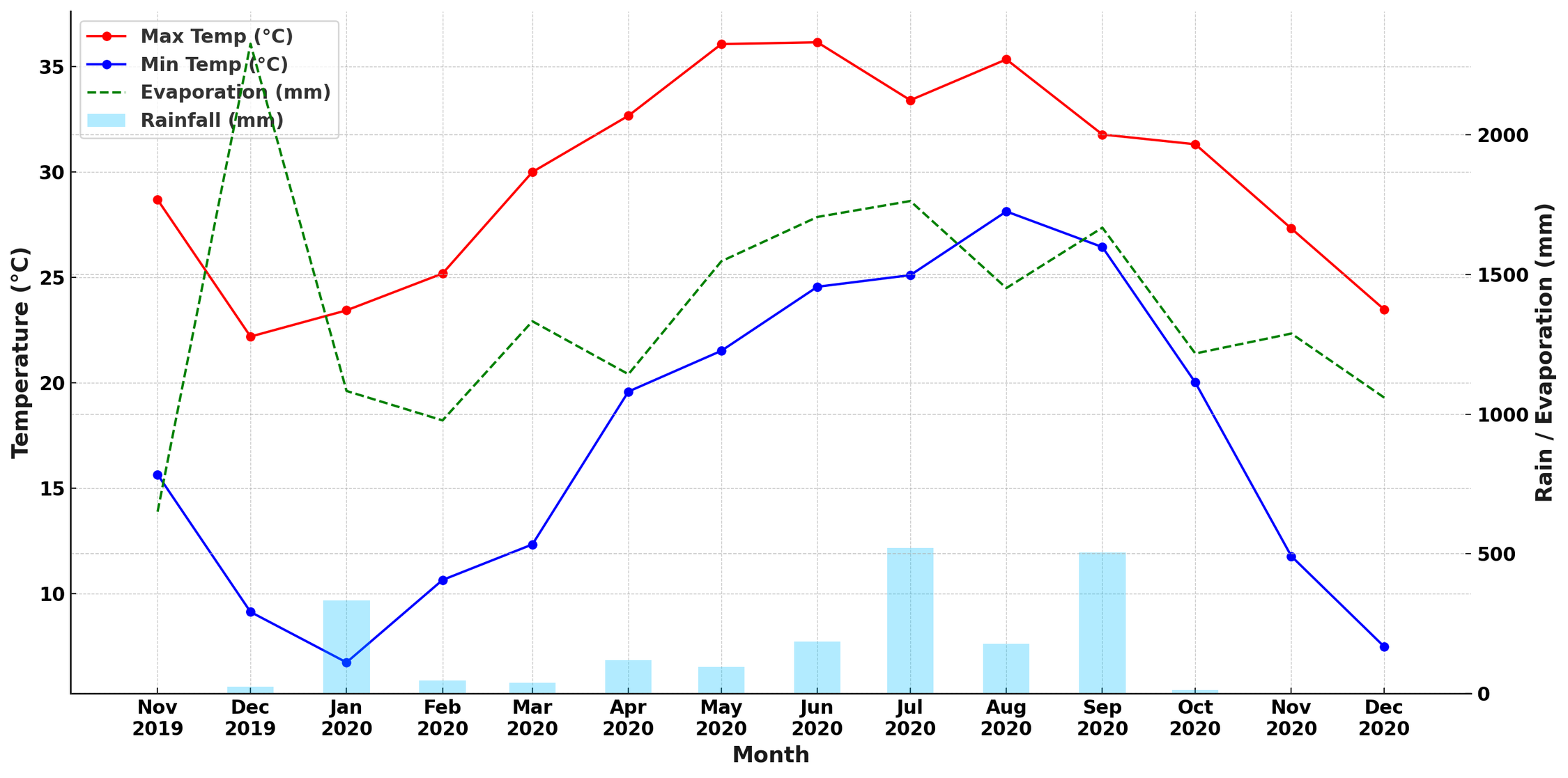

Supplement: S1 Fig — (TIF) [file pone.0334042.s001.tif]
