# Supplementary material for: Nutrient synergy in wheat: Impacts of nitrogen and boron on productivity, accumulation, and soil nutrient retention
Source: PLoS One. 2025 Oct 6;20(10):e0334042. doi: 10.1371/journal.pone.0334042 (PMC12500113; doi:10.1371/journal.pone.0334042)
Supplement: S1 Table — The table includes average maximum and minimum air temperature (°C), total rainfall (mm), and total evaporation (mm) recorded for each month. (DOCX) [file pone.0334042.s002.docx]

**Table S1. Monthly weather data for the study area from November 2019 to December 2020. The table includes average maximum and minimum air temperature (°C), total rainfall (mm), and total evaporation (mm) recorded for each month.**

| **Year** | **Months** | **Max (^0^C)** | **MIN (^0^C)** | **Rain (mm)** | **Evaporation (mm)** |
| --- | --- | --- | --- | --- | --- |
| **2019** | November | 28.68667 | 15.65333 | 0 | 650.59 |
| **2019** | December | 22.19355 | 9.132258 | 24.8 | 2325.79 |
| **2020** | January | 23.43871 | 6.73871 | 33.7 | 1082.81 |
| **2020** | Feburary | 25.18571 | 10.65 | 47.2 | 977.49 |
| **2020** | March | 30 | 12.33548 | 37.6 | 1332.3 |
| **2020** | April | 32.67 | 19.58667 | 119.1 | 1142.78 |
| **2020** | May | 36.06774 | 21.51935 | 94.4 | 1547.13 |
| **2020** | June | 36.15333 | 24.55667 | 186.2 | 1705.71 |
| **2020** | July | 33.40645 | 25.10742 | 519.7 | 1762.79 |
| **2020** | August | 35.34194 | 28.12903 | 176.8 | 1451.05 |
| **2020** | September | 31.78 | 26.44 | 504.2 | 1667.44 |
| **2020** | October | 31.31613 | 20.01613 | 12.4 | 1216.8 |
| **2020** | November | 27.31933 | 11.77667 | 0 | 1288.38 |
| **2020** | December | 23.47742 | 7.483871 | 0 | 1058.88 |
