# Supplementary material for: Nutrient synergy in wheat: Impacts of nitrogen and boron on productivity, accumulation, and soil nutrient retention
Source: PLoS One. 2025 Oct 6;20(10):e0334042. doi: 10.1371/journal.pone.0334042 (PMC12500113; doi:10.1371/journal.pone.0334042)
Supplement: S2 Table — (DOCX) [file pone.0334042.s003.docx]

**Table S2. Summary statistics (mean, standard deviation, and standard error) of yields attributing traits under different N and B treatments.**

|  |  | **Effective tillers (m^-2^)** | | | | **Sterility (%)** | | | | **Kernel per spike** | | | | **1000 grain weight (g)** | | | **Spike length (cm)** | | | **Spike weight (g)** | | |
| --- | --- | --- | --- | --- | --- | --- | --- | --- | --- | --- | --- | --- | --- | --- | --- | --- | --- | --- | --- | --- | --- | --- |
| **Factor A** | **Factor B** | **Mean** | **SD** | **SE** | **Mean** | | **SD** | **SE** | **Mean** | | **SD** | **SE** | **Mean** | | **SD** | **SE** | **Mean** | **SD** | **SE** | **Mean** | **SD** | **SE** |
| **N0** | **B0** | 253.33 | 62.52 | 36.09 | 53.09 | | 4.36 | 2.52 | 22.00 | | 1.78 | 1.03 | 43.50 | | 2.66 | 1.53 | 8.00 | 0.69 | 0.40 | 1.97 | 0.01 | 0.01 |
| **N0** | **B1** | 280.00 | 18.03 | 10.41 | 44.35 | | 2.86 | 1.65 | 24. 40 | | 2.42 | 1.40 | 44.95 | | 2.18 | 1.26 | 7.66 | 0.26 | 0.15 | 1.98 | 0.02 | 0.01 |
| **N0** | **B2** | 280.00 | 22.91 | 13.23 | 34.49 | | 4.83 | 2.79 | 28.13 | | 2.48 | 1.43 | 44.00 | | 0.81 | 0.47 | 8.14 | 0.33 | 0.19 | 2.01 | 0.03 | 0.02 |
| **N1** | **B0** | 333.33 | 10.41 | 6.01 | 56.38 | | 3.22 | 1.86 | 24.73 | | 6.57 | 3.80 | 47.28 | | 2.01 | 1.16 | 9.06 | 0.54 | 0.31 | 1.97 | 0.02 | 0.01 |
| **N1** | **B1** | 333.33 | 28.43 | 16.41 | 39.74 | | 8.35 | 4.82 | 27.53 | | 2.50 | 1.44 | 47.29 | | 1.95 | 1.12 | 9.02 | 0.09 | 0.05 | 2.07 | 0.05 | 0.03 |
| **N1** | **B2** | 323.33 | 41.93 | 24.21 | 33.34 | | 7.50 | 4.33 | 31.73 | | 5.26 | 3.04 | 44.49 | | 1.99 | 1.15 | 8.93 | 0.11 | 0.06 | 2.15 | 0.04 | 0.02 |
| **N2** | **B0** | 368.33 | 22.55 | 13.02 | 54.43 | | 5.81 | 3.36 | 23.40 | | 4.69 | 2.71 | 48.64 | | 4.15 | 2.40 | 9.15 | 0.60 | 0.35 | 2.13 | 0.05 | 0.03 |
| **N2** | **B1** | 356.67 | 32.15 | 18.56 | 38.98 | | 4.94 | 2.85 | 27.67 | | 1.53 | 0.88 | 45.65 | | 0.32 | 0.19 | 9.64 | 0.30 | 0.17 | 2.25 | 0.01 | 0.01 |
| **N2** | **B2** | 336.67 | 41.63 | 24.04 | 35.32 | | 6.89 | 3.98 | 29.27 | | 3.67 | 2.12 | 46.68 | | 1.35 | 0.78 | 9.93 | 0.44 | 0.25 | 2.28 | 0.02 | 0.01 |
| **N3** | **B0** | 401.67 | 40.10 | 23.15 | 56.56 | | 4.25 | 2.45 | 20.00 | | 0.00 | 0.00 | 54.03 | | 2.60 | 1.50 | 10.08 | 0.17 | 0.10 | 2.25 | 0.01 | 0.01 |
| **N3** | **B1** | 391.67 | 31.75 | 18.33 | 42.41 | | 6.46 | 3.73 | 28.87 | | 6.52 | 3.77 | 51.68 | | 3.64 | 2.10 | 10.01 | 0.84 | 0.48 | 2.36 | 0.04 | 0.02 |
| **N3** | **B2** | 408.33 | 27.54 | 15.90 | 35.47 | | 4.80 | 2.77 | 29.27 | | 0.70 | 0.41 | 52.27 | | 1.22 | 0.70 | 10.07 | 0.86 | 0.50 | 2.42 | 0.04 | 0.02 |
