# Supplementary material for: Nutrient synergy in wheat: Impacts of nitrogen and boron on productivity, accumulation, and soil nutrient retention
Source: PLoS One. 2025 Oct 6;20(10):e0334042. doi: 10.1371/journal.pone.0334042 (PMC12500113; doi:10.1371/journal.pone.0334042)
Supplement: S3 Table — (DOCX) [file pone.0334042.s004.docx]

**Table S3. Summary statistics (mean, standard deviation, and standard error) of yields under different N and B treatments.**

|  |  | **Grain yield (t ha^-1^)** | | | **Straw dry matter (t ha^-1^)** | | | **B:C ratio** | | |
| --- | --- | --- | --- | --- | --- | --- | --- | --- | --- | --- |
| **Factor A** | **Factor B** | **Mean** | **SD** | **SE** | **Mean** | **SD** | **SE** | **Mean** | **SD** | **SE** |
| **N0** | **B0** | 3.93 | 1.04 | 0.60 | 4.07 | 0.16 | 0.09 | 1.71 | 0.45 | 0.26 |
| **N0** | **B1** | 4.31 | 0.86 | 0.50 | 3.92 | 0.06 | 0.03 | 1.83 | 0.36 | 0.21 |
| **N0** | **B2** | 4.92 | 1.24 | 0.71 | 3.95 | 0.10 | 0.06 | 2.02 | 0.51 | 0.29 |
| **N1** | **B0** | 4.28 | 0.74 | 0.43 | 4.26 | 0.27 | 0.15 | 1.80 | 0.31 | 0.18 |
| **N1** | **B1** | 5.14 | 1.60 | 0.92 | 4.16 | 0.24 | 0.13 | 2.11 | 0.65 | 0.38 |
| **N1** | **B2** | 5.46 | 1.58 | 0.91 | 4.28 | 0.52 | 0.30 | 2.17 | 0.62 | 0.36 |
| **N2** | **B0** | 5.02 | 0.54 | 0.31 | 4.44 | 0.56 | 0.32 | 2.11 | 0.22 | 0.13 |
| **N2** | **B1** | 5.46 | 0.96 | 0.55 | 4.68 | 0.49 | 0.28 | 2.22 | 0.39 | 0.22 |
| **N2** | **B2** | 6.17 | 0.96 | 0.55 | 4.84 | 0.53 | 0.30 | 2.42 | 0.37 | 0.21 |
| **N3** | **B0** | 5.42 | 0.76 | 0.44 | 5.35 | 0.21 | 0.12 | 2.52 | 0.31 | 0.18 |
| **N3** | **B1** | 5.83 | 0.59 | 0.34 | 5.78 | 0.27 | 0.15 | 2.34 | 0.24 | 0.13 |
| **N3** | **B2** | 6.26 | 1.63 | 0.94 | 5.84 | 0.52 | 0.30 | 2.82 | 0.63 | 0.36 |
