# Supplementary material for: Nutrient synergy in wheat: Impacts of nitrogen and boron on productivity, accumulation, and soil nutrient retention
Source: PLoS One. 2025 Oct 6;20(10):e0334042. doi: 10.1371/journal.pone.0334042 (PMC12500113; doi:10.1371/journal.pone.0334042)
Supplement: S4 Table — (DOCX) [file pone.0334042.s005.docx]

**Table S4. Summary statistics (mean, standard deviation, and standard error) of N content and uptake in grain and straw under different N and B treatments.**

|  |  | **Grain N (g kg^-1^)** | | | **Grain protein (%)** | | | **Grain N uptake (kg ha^-1^)** | | | **Straw N (g kg^-1^)** | | | **Straw N uptake (kg ha^-1^)** | | |
| --- | --- | --- | --- | --- | --- | --- | --- | --- | --- | --- | --- | --- | --- | --- | --- | --- |
| **Factor A** | **Factor B** | **Mean** | **SD** | **SE** | **Mean** | **SD** | **SE** | **Mean** | **SD** | **SE** | **Mean** | **SD** | **SE** | **Mean** | **SD** | **SE** |
| **N0** | **B0** | 8.48 | 2.05 | 1.18 | 5.03 | 1.28 | 0.74 | 32.07 | 4.22 | 2.4 | 2.32 | 0.26 | 0.15 | 11.68 | 1.46 |  |
| **N0** | **B1** | 7.78 | 0.98 | 0.56 | 4.86 | 0.61 | 0.35 | 33.26 | 5.70 | 3.29 | 2.56 | 0.26 | 0.15 | 14.43 | 5.21 | 0.84 |
| **N0** | **B2** | 9.33 | 0.64 | 0.37 | 5.83 | 0.40 | 0.23 | 46.44 | 14.40 | 8.31 | 2.26 | 0.20 | 0.12 | 12.69 | 2.81 | 3.00 |
| **N1** | **B0** | 10.84 | 1.36 | 0.78 | 6.77 | 0.85 | 0.49 | 45.33 | 4.12 | 2.38 | 2.60 | 0.20 | 0.11 | 16.90 | 6.37 | 1.62 |
| **N1** | **B1** | 11.75 | 1.20 | 0.69 | 7.34 | 0.75 | 0.43 | 60.71 | 21.61 | 12.74 | 2.63 | 0.23 | 0.13 | 15.74 | 3.34 | 3.67 |
| **N1** | **B2** | 11.75 | 0.94 | 0.54 | 7.33 | 0.59 | 0.34 | 65.04 | 22.92 | 13.23 | 2.23 | 0.23 | 0.13 | 14.65 | 6.13 | 1.93 |
| **N2** | **B0** | 13.66 | 0.07 | 0.04 | 8.53 | 0.04 | 0.02 | 68.63 | 7.74 | 4.47 | 3.10 | 0.45 | 0.26 | 24.70 | 10.67 | 3.54 |
| **N2** | **B1** | 13.46 | 0.15 | 0.08 | 8.41 | 0.09 | 0.05 | 73.54 | 12.17 | 7.02 | 3.33 | 0.47 | 0.27 | 22.30 | 3.31 | 6.16 |
| **N2** | **B2** | 12.90 | 0.6 | 0.34 | 8.06 | 0.37 | 0.21 | 79.94 | 15.92 | 9.19 | 3.10 | 1.05 | 0.61 | 20.67 | 6.48 | 1.91 |
| **N3** | **B0** | 14.85 | 0.75 | 0.43 | 9.28 | 0.47 | 0.27 | 80.14 | 7.73 | 4.46 | 4.46 | 0.90 | 0.52 | 35.46 | 13.90 | 8.02 |
| **N3** | **B1** | 15.54 | 0.98 | 0.56 | 9.71 | 0.61 | 0.35 | 90.29 | 4.28 | 2.47 | 5.06 | 0.66 | 0.38 | 35.37 | 12.79 | 7.38 |
| **N3** | **B2** | 13.03 | 0.46 | 0.26 | 8.14 | 0.28 | 0.16 | 95.15 | 24.13 | 13.93 | 5.30 | 0.95 | 0.55 | 38.15 | 9.62 | 5.55 |
