# Supplementary material for: Nutrient synergy in wheat: Impacts of nitrogen and boron on productivity, accumulation, and soil nutrient retention
Source: PLoS One. 2025 Oct 6;20(10):e0334042. doi: 10.1371/journal.pone.0334042 (PMC12500113; doi:10.1371/journal.pone.0334042)
Supplement: S5 Table — (DOCX) [file pone.0334042.s006.docx]

|  |  | **Grain B content (mg kg^-1^)** | | | **Straw B content (mg kg^-1^)** | | | **Grain B uptake (kg ha^-1^) X 10^-5^** | | | **Straw B uptake (kg ha^-1^) X 10^-5^** | | |
| --- | --- | --- | --- | --- | --- | --- | --- | --- | --- | --- | --- | --- | --- |
| **Factor A** | **Factor B** | **Mean** | **SD** | **SE** | **Mean** | **SD** | **SE** | **Mean** | **SD** | **SE** | **Mean** | **SD** | **SE** |
| **N0** | **B0** | 2.0 | 0.61 | 0.35 | 11.58 | 0.36 | 0.21 | 0.75 | 0.19 | 0.11 | 5.97 | 1.30 | 0.75 |
| **N0** | **B1** | 4.4 | 0.62 | 0.36 | 12.43 | 0.19 | 0.11 | 1.89 | 0.45 | 0.26 | 7.19 | 2.7 | 1.56 |
| **N0** | **B2** | 5.06 | 0.14 | 0.08 | 12.75 | 0.36 | 0.21 | 2.50 | 0.69 | 0.39 | 7.11 | 1.22 | 0.70 |
| **N1** | **B0** | 1.65 | 0.30 | 0.17 | 11.70 | 0.59 | 0.34 | 0.68 | 0.028 | 0.016 | 7.46 | 1.93 | 1.11 |
| **N1** | **B1** | 4.40 | 0.49 | 0.28 | 12.56 | 0.065 | 0.03 | 2.24 | 0.59 | 0.34 | 7.50 | 1.47 | 0.84 |
| **N1** | **B2** | 5.19 | 0.11 | 0.06 | 13.00 | 0.35 | 0.20 | 2.83 | 0.77 | 0.44 | 8.04 | 3.13 | 1.81 |
| **N2** | **B0** | 2.30 | 0.06 | 0.35 | 12.11 | 0.20 | 0.12 | 1.15 | 0.096 | 0.055 | 9.41 | 2.89 | 1.67 |
| **N2** | **B1** | 4.21 | 0.24 | 0.13 | 12.69 | 0.28 | 0.17 | 2.31 | 0.51 | 0.29 | 8.52 | 0.75 | 0.43 |
| **N2** | **B2** | 4.88 | 0.25 | 0.14 | 13.21 | 0.28 | 0.17 | 3.02 | 0.48 | 0.28 | 8.95 | 1.59 | 0.91 |
| **N3** | **B0** | 1.52 | 0.38 | 0.22 | 11.64 | 0.21 | 0.12 | 0.84 | 0.32 | 0.19 | 9.01 | 2.30 | 1.33 |
| **N3** | **B1** | 4.22 | 0.66 | 0.37 | 12.26 | 0.22 | 0.13 | 2.46 | 0.43 | 0.24 | 8.40 | 2.20 | 1.27 |
| **N3** | **B2** | 4.98 | 0.31 | 0.17 | 12.67 | 0.30 | 0.17 | 2.31 | 0.82 | 0.47 | 9.05 | 0.63 | 0.36 |

# Table S5. Summary statistics (mean, standard deviation, and standard error) of B content and uptake in grain and straw under different N and B treatments.
