# Supplementary material for: Nutrient synergy in wheat: Impacts of nitrogen and boron on productivity, accumulation, and soil nutrient retention
Source: PLoS One. 2025 Oct 6;20(10):e0334042. doi: 10.1371/journal.pone.0334042 (PMC12500113; doi:10.1371/journal.pone.0334042)
Supplement: S6 Table — (DOCX) [file pone.0334042.s007.docx]

**Table S6. Summary statistics (mean, standard deviation, and standard error) of post harvest soil properties under different N and B treatments.**

|  |  | **pH** | | | **OM (%)** | | | **P_2_O_5_ (kg ha^-1)^** | | | **K_2_O (kg ha^-1)^** | | | **N (%)** | | | **B (mg kg^-1^)** | | |
| --- | --- | --- | --- | --- | --- | --- | --- | --- | --- | --- | --- | --- | --- | --- | --- | --- | --- | --- | --- |
| **Factor A** | **Factor B** | **Mean** | **SD** | **SE** | **Mean** | **SD** | **SE** | **Mean** | **SD** | **SE** | **Mean** | **SD** | **SE** | **Mean** | **SD** | **SE** | **Mean** | **SD** | **SE** |
| **N0** | **B0** | 6.26 | 0.54 | 0.31 | 1.98 | 0.21 | 0.12 | 73.49 | 2.55 | 1.47 | 317.20 | 75.53 | 43.61 | 0.06 | 0.01 | 0.01 | 0.15 | 0.04 | 0.02 |
| **N0** | **B1** | 6.77 | 0.57 | 0.33 | 1.90 | 0.14 | 0.08 | 74.22 | 3.22 | 1.86 | 309.20 | 51.27 | 29.60 | 0.07 | 0.02 | 0.01 | 0.36 | 0.09 | 0.05 |
| **N0** | **B2** | 6.06 | 0.28 | 0.16 | 2.18 | 0.11 | 0.06 | 72.29 | 0.70 | 0.41 | 286.00 | 32.76 | 18.91 | 0.07 | 0.01 | 0.01 | 0.54 | 0.03 | 0.01 |
| **N1** | **B0** | 6.16 | 0.22 | 0.13 | 1.90 | 0.12 | 0.07 | 73.29 | 2.53 | 1.46 | 286.00 | 76.74 | 44.30 | 0.08 | 0.00 | 0.00 | 0.21 | 0.06 | 0.04 |
| **N1** | **B1** | 6.44 | 0.82 | 0.48 | 1.92 | 0.54 | 0.31 | 72.69 | 1.03 | 0.59 | 321.87 | 131.76 | 76.07 | 0.10 | 0.01 | 0.01 | 0.30 | 0.06 | 0.04 |
| **N1** | **B2** | 6.21 | 0.30 | 0.17 | 2.14 | 0.20 | 0.12 | 73.49 | 0.76 | 0.44 | 270.80 | 63.92 | 36.90 | 0.09 | 0.01 | 0.01 | 0.58 | 0.19 | 0.11 |
| **N2** | **B0** | 5.71 | 0.03 | 0.02 | 1.92 | 0.09 | 0.05 | 71.62 | 0.69 | 0.40 | 306.00 | 20.78 | 12.00 | 0.11 | 0.03 | 0.02 | 0.13 | 0.09 | 0.05 |
| **N2** | **B1** | 6.41 | 0.31 | 0.18 | 1.93 | 0.29 | 0.17 | 75.02 | 4.04 | 2.33 | 318.80 | 98.85 | 57.07 | 0.13 | 0.06 | 0.03 | 0.32 | 0.12 | 0.07 |
| **N2** | **B2** | 6.18 | 0.71 | 0.41 | 1.93 | 0.10 | 0.06 | 74.15 | 1.60 | 0.93 | 382.80 | 79.56 | 45.94 | 0.11 | 0.04 | 0.03 | 0.56 | 0.13 | 0.07 |
| **N3** | **B0** | 6.38 | 0.37 | 0.21 | 2.03 | 0.80 | 0.46 | 75.55 | 2.69 | 1.55 | 370.00 | 34.97 | 20.19 | 0.12 | 0.03 | 0.02 | 0.17 | 0.04 | 0.02 |
| **N3** | **B1** | 6.11 | 0.30 | 0.17 | 1.91 | 0.22 | 0.13 | 75.36 | 4.25 | 2.45 | 347.60 | 42.28 | 24.41 | 0.15 | 0.02 | 0.01 | 0.31 | 0.06 | 0.03 |
| **N3** | **B2** | 6.15 | 0.46 | 0.26 | 1.99 | 0.35 | 0.20 | 74.69 | 4.64 | 2.68 | 280.40 | 86.04 | 49.68 | 0.18 | 0.01 | 0.01 | 0.75 | 0.28 | 0.16 |
